# The Effects of Fecal Microbiota Transplantation on the Symptoms and the Duodenal Neurogenin 3, Musashi 1, and Enteroendocrine Cells in Patients With Diarrhea-Predominant Irritable Bowel Syndrome

**DOI:** 10.3389/fcimb.2021.524851

**Published:** 2021-05-12

**Authors:** Tarek Mazzawi, Magdy El-Salhy, Gülen Arslan Lied, Trygve Hausken

**Affiliations:** ^1^ Division of Gastroenterology, Department of Medicine, Haukeland University Hospital, Bergen, Norway; ^2^ National Center for Functional Gastrointestinal Disorders, Division of Gastroenterology, Haukeland University Hospital, Bergen, Norway; ^3^ Center for Nutrition, Department of Clinical Medicine, University of Bergen, Bergen, Norway; ^4^ Division of Gastroenterology, Department of Medicine, Stord Hospital, Helse-Fonna, Stord, Norway

**Keywords:** cell densities, duodenum, neuroendocrine, fecal microbiota transplantation, gut hormones, microbiota, irritable bowel syndrome, stem cells

## Abstract

**Introduction:**

Interactions between the gut microbiota and enteroendocrine cells play important role in irritable bowel syndrome (IBS). Reduced stem cell densities and their differentiation into enteroendocrine cells may cause abnormal densities of the duodenal enteroendocrine cells in IBS patients.

**Materials and Methods:**

We aimed to investigate the effects of fecal microbiota transplantation (FMT) on stem cell differentiation into enteroendocrine cells as detected by neurogenin 3, stem cells as detected by Musashi 1, and the enteroendocrine cells in the duodenum of IBS patients. The study included 16 IBS patients according to Rome III criteria. Four patients were excluded. The remaining patients (n = 12, four females and eight males) were divided according to the cause of IBS into post-infectious (*n* = 6) and idiopathic (*n* = 6) IBS. They completed the following questionnaires before and 3 weeks after FMT: IBS-Symptom Severity Scoring system (IBS-SSS) and IBS-Symptom Questionnaire (IBS-SQ). Feces donated by healthy relatives of the patients were transplanted *via* gastroscope. Biopsies were taken from the descending part of the duodenum at baseline and 3 weeks after FMT. They were immunostained for neurogenin 3, Musashi 1, and all types of duodenal enteroendocrine cells and quantified by computerized image analysis. Microbiota analyses of feces collected just before and 3 weeks after FMT were performed using GA-map™ Dysbiosis test (Genetic Analysis AS, Oslo, Norway).

**Results:**

The total scores for IBS-SSS and IBS-SQ were significantly improved 3 weeks after receiving FMT, *P* = 0.0009 and <0.0001, respectively. The stem cell densities of neurogenin 3 increased significantly following FMT (*P* = 0.0006) but not for Musashi 1 (*P* = 0.42). The cell densities of chromogranin A, cholecystokinin, gastric inhibitory peptide, serotonin, and somatostatin, but not for secretin, have significantly changed in both IBS groups after 3 weeks from receiving FMT.

**Conclusion:**

More than two-thirds of IBS patients experienced improvement in their symptoms parallel to changes in the enteroendocrine cells densities 3 weeks after FMT. The changes in the enteroendocrine cell densities do not appear to be caused by changes in the stem cells or their early progenitors rather by changes in the differentiation progeny as detected by neurogenin 3. The study was retrospectively registered at ClinicalTrials.gov (ID: NCT03333291).

**Clinical Trial Registration:**

ClinicalTrials.gov, identifier NCT03333291.

## Introduction

The neuroendocrine peptides/amines produced by the enteroendocrine cells regulate several functions of the gastrointestinal (GI) tract such as motility, secretion, absorption, visceral sensitivity, local immune-defense, cell proliferation, and appetite (El-Salhy et al., 2014; El-Salhy et al., 2017). The enteroendocrine cells possess sensory tentacle protruding into the intestinal lumen that sense the changes in the intestinal lumen and respond by releasing their neuroendocrine peptides/amines (Sternini et al., 2008; El-Salhy et al., 2016). Enteroendocrine cells, stem cells, and their progenitor cells are abnormal in the proximal and distal parts of the small intestine of patients with irritable bowel syndrome (IBS) (Dizdar et al., 2010; El-Salhy et al., 2010; El-Salhy et al., 2014a; El-Salhy, 2015; El-Salhy and Gilja, 2017). The abnormalities in enteroendocrine cells seem to explain the dysmotility, visceral hypersensitivity, and abnormal intestinal secretion seen in IBS patients (El-Salhy et al., 2014).

Patients with IBS also have an altered gut microbiota profile (Carroccio et al., 2010; Ohman and Simren, 2013; Hong and Rhee, 2014) *i.e.*; fewer *Lactobacillus* and *Bifidobacterium* spp. and more *Clostridium* spp. that ferment fibers and fermentable oligosaccharides, disaccharides and monosaccharides, and polyols (FODMAPs), and produce gas leading to luminal distension with symptoms of the abdominal pain and bloating (Si et al., 2004; Kassinen et al., 2007; El-Salhy et al., 2014b). Probiotics (live bacterial cultures) have been used to alleviate the symptoms of IBS (Sisson et al., 2014), but unfortunately, they have shown limited effectiveness (Malikowski et al., 2017). Human feces from a healthy subject are used to treat patients with recurrent *Clostridium difficile* colitis through re-establishing the balance in the gut microbiota (van Nood et al., 2013). A new concept is emerging where FMT is used as a treatment option for IBS symptoms (Pinn et al., 2014; Halkjaer et al., 2018; Johnsen et al., 2018; Mazzawi et al., 2018; El-Salhy et al., 2019).

The gut microbiota affect both the enteroendocrine cells and the enteric nervous system (Reigstad et al., 2015; Greiner and Backhed, 2016). A published study by our group (Mazzawi et al., 2018) has shown that using FMT in IBS patients reduces their symptoms, improves their quality of life within the first week, and restores the imbalance in the gut microbiota within 3 weeks, which lasts for 28 weeks after receiving FMT. The mechanisms behind this improvement are still unknown. The aim of this study was to investigate whether the improvement brought about by FMT in these IBS patients was caused by the interaction of the microbiota with the stem or/and enteroendocrine cells. Thus, the possible changes in the densities of Musashi 1 (Msi-1, a marker for stem cells and their early progenitors) (Bjerknes and Cheng, 2006), neurogenin 3 (NEUROG3, a marker for enteroendocrine cells progenitor) (Montgomery and Breault, 2008), and enteroendocrine cells in IBS patients following FMT were studied.

## Materials and Methods

### Patients, Donors, and Controls

A recipient group of 16 IBS patients who met the Rome III criteria for IBS and were referred to the gastroenterology outpatient clinic, Haukeland University Hospital, Bergen, Norway during the year of 2015 were included in the study. The patients had moderate to severe abdominal symptoms as defined by IBS-Symptom Severity Scoring system (IBS-SSS) score >175. The exclusion criteria included history of inflammatory bowel diseases, GI malignancy, blood in stool, immunocompromised patients who are taking immunosuppressive medications, history of opportunistic infections within 1 year prior to FMT, oral thrush, or disseminated lymphadenopathy. Patients who were scheduled for abdominal surgery, pregnant or lactating women, and patients taking probiotics or antibiotics within 1 month prior to FMT were also excluded.

A donor group of healthy family members, seven males and nine females with an age range 20–55 (mean age 35) years were also included. The exclusion criteria of the donors were pregnancy, history of inflammatory bowel diseases, IBS, chronic abdominal pain, GI malignancy, diarrhea, blood in stool, antibiotic and probiotic use within 1 month prior to FMT, immunocompromised patients who were consuming immunosuppressive medications, history of opportunistic infections within 1 year prior to FMT, oral thrush, and disseminated lymphadenopathy.

Biopsy samples from a group of 12 healthy volunteers, 10 females and two males with an age range 20–42 (mean age 39) years, recruited at Stord Hospital, Stord, Norway by advertising in the local newspapers, were used as controls to study duodenal stem cells and enteroendocrine cells.

The study was performed in accordance with the Declaration of Helsinki (World Medical Association Declaration of Helsinki, 2013), adhered to CONSORT guidelines and was approved by the Regional Committee for Medical and Health Research Ethics in Western Norway (reference no.: 2013/1497). The current study was retrospectively registered on 03.Nov.2017 at ClinicalTrials.gov (ID: NCT03333291). All of the participants provided written informed consent.

### Study Design

The donors and patients were screened one week before FMT according to the reviews and guidelines that were published prior to the start of the study in the year 2015 (Aroniadis and Brandt, 2013; Cammarota et al., 2014). They were physically examined, and blood tests were taken to investigate previous exposure to contagious infectious agents including serologic testing for the donors (hepatitis A, B and C, HIV, Epstein–Barr virus and cytomegalovirus) and for the patients [hemoglobin, leucocytes, platelets, creatinine, aspartate aminotransferase, alanine aminotransferase, International normalized ratio (INR), chromogranin A (CgA), and electrolytes]. Stool samples from both donors and patients were examined for fecal calprotectin, cultured for enteric bacterial pathogens, and screened for viruses, parasites, and eggs. The patients were told not to change their lifestyle or diet before the start or during the study and to report any changes in their lifestyle or use of new medications during the study.

### FMT Procedure

On transplantation day, 30 g of fresh donors’ feces (<2 h from production to donation) was mixed with 60 ml normal saline for preparing the fecal suspension using manual effort and sieved in order to avoid the clogging of infusion syringes and tubes (Aroniadis and Brandt, 2013; Cammarota et al., 2014). All of the patients fasted overnight and received only once a 60 ml of fecal suspension followed by 60 ml normal saline, using a 60 ml syringe, infused through the gastroscope into the descending part of the duodenum past the duodenal papilla at a steady rate within 7–10 seconds to avoid a fast flush and regurgitation of fecal material into the stomach. Tissue biopsies were taken from the descending part of the duodenum before installation of feces and again 3 weeks after FMT. Fecal samples were collected on the same day of FMT (before the procedure) and 3 weeks after FMT and sent for microbiota analysis using GA-map™ Dysbiosis test (Genetic Analysis AS, Oslo, Norway) as previously explained in details (Mazzawi et al., 2018).

### Questionnaires

The patients completed the following questionnaires before and 3 weeks after FMT for IBS symptoms assessment: a) IBS-Symptom Severity Scoring system (IBS-SSS), in which higher scores indicated worse symptoms (scores <175 represented mild IBS symptoms, 175–300 moderate severity, and >300 severe IBS) and a decrease of 50 points correlated with improvement in clinical symptoms (Francis et al., 1997), and b) IBS symptom questionnaire (IBS-SQ) reported IBS symptoms (nausea, bloating, abdominal pain, constipation, diarrhea, and anorexia) using a severity scale from 0 to 10, where 0 = no symptoms and 10 = severe symptoms (Mathias et al., 1994; Kane et al., 2003).

### Gastroscopy and Immunohistochemistry

The patients fasted overnight prior to undergoing a gastroscopy. During the gastroscopy, four tissue biopsies were collected from the descending part of the duodenum, distal to the papilla. The biopsy samples were fixed in 4% buffered paraformaldehyde, paraffin-embedded biopsies were cut into 5 μm thick sections. The sections were then stained with hematoxylin–eosin and immunostained with an ultraView Universal DAB Detection Kit (cat.no. 760-500, Ventana Medical Systems, Basal, Switzerland) and the BenchMark Ultra IHC/ISH staining module (Ventana Medical Systems). The sections were incubated with the primary antibodies for 32 min at 37°C. The primary antibodies that were diluted according to the suppliers’ recommendations were as follows: polyclonal rabbit anti-synthetic peptide conjugated to keyhole limpet hemocyanin derived from within residues 1–100 of human Msi-1 (code ab21628, Abcam, Cambridge, United Kingdom), monoclonal mouse-anti-protein expressed in 293T cells transfected with human NEUROG3 expression vector (code ab87108, Abcam), monoclonal mouse antibody raised against the N-terminal of purified CgA (code no. M869, Dako, Glostrup, Denmark), polyclonal rabbit anti-human secretin for the detection of secretin (code no. sc-20938, Santa Cruz Biotechnology, Santa Cruz, CA, United States), rabbit antibodies for the detection of cholecystokinin (CCK; code no. A0568, Dako, Glostrup, Denmark), mouse antibodies against human synthetic gastric inhibitory peptide (GIP; code no. sc-57162, Santa Cruz Biotechnology), mouse antibodies against serotonin (code no. R87104 B56-1, Dako), and rabbit antibodies against synthetic cyclic somatostatin (code no. A0566, Dako). Labeled secondary antibodies locate the specific antibody. The complex is then visualized with hydrogen peroxide substrate and 3, 3′-diaminobenzidine tetrahydrochloride (DAB) chromogen, which produces a brown precipitate, and counterstained with hematoxylin.

### Computerized Image Analysis

1) The densities of Msi-1 and NEUROG3 were quantified using ×40 objective on the light microscope equipped with a digital camera (DP 26, Olympus, Tokyo, Japan) connected to a computer equipped with a software cellSens imaging program (version 1.7, Olympus, Tokyo, Japan). The number of immunoreactive cells and crypts was quantified in 10 randomly chosen fields. Each field (frame) represented a tissue area of 0.035 mm^2^. Msi-1 cell density is expressed as the number of cells/10 crypts and the density of NEUROG3 as number of cells/field.

2) Enteroendocrine cell densities were quantified using a light microscope with ×40 objective and a computer software Cell^B imaging program (Olympus, Tokyo, Japan). The number of immunoreactive cells was quantified in 10 randomly chosen fields. Each field (frame) of epithelial cells represents a tissue area measured at 0.09 mm^2^. The density of each enteroendocrine cell type was expressed as the number of cells/mm^2^ of epithelium.

By keeping the identity of the slides concealed, the quantification of Msi-1 and NEUROG3 was performed by M.E.S., and the quantification of the enteroendocrine cells was performed by T.M.

### Gut Microbiota Analysis

The gut microbiota analysis was previously described in details (Mazzawi et al., 2018). Briefly, GA-map™ Dysbiosis test is based on fecal homogenization, mechanical bacterial cell disruption, and automated total bacterial genomic DNA extraction using magnetic beads. Fifty-four DNA probes were used targeting more than 300 bacterial strains based on their 16S rRNA sequence in seven variable regions (V3–V9). Twenty-six bacteria probes are species specific, 19 detect bacteria on the genus level, and nine probes detect bacteria at higher taxonomic levels. Probe labeling is by single nucleotide extension and hybridization to complementary probes coupled to magnetic beads and signal detection by using BioCode 1000A 128-Plex Analyzer (Applied BioCode, Santa Fe Springs, CA, USA) (Casen et al., 2015).

### Statistical Analysis

Kruskal–Wallis with Dunn’s *post hoc* test was used to compare the cell densities between the controls and the patients before and after receiving FMT. Paired *t-*test is used to compare between the cell densities of the patients before and after receiving FMT. Mann–Whitney U test was used to compare the microbiota profiles of the patients before and after FMT to their respective donors. Correlations were performed using Spearman non-parametric test. The data are presented as mean ± SEM values. *P <*0.05 is considered to be statistically significant.

## Results

### Patients

Four out of the 16 originally included patients were excluded after withdrawing their consents to participate for practical reasons (*n* = 1), failed gastroscope intubation after FMT (*n* = 1), being diagnosed with functional dyspepsia (*n* = 1), and positive stool culture for *Clostridium difficile* (*n* = 1). The remaining patients (*n* = 12) were four females and eight males, with age range 20–44 years. Six patients suffered from post-infectious IBS (PI-IBS), and six had idiopathic IBS. They completed the whole study by filling out the questionnaires and delivered stools for microbiota analysis at the same day of undergoing gastroscopy with duodenal biopsies *i.e.* the day of FMT and after 3 weeks. No change in lifestyle, diet, or use of any/new medications has been registered during the study.

### Questionnaires

Three weeks following FMT, the symptoms of IBS as assessed by IBS-SSS improved in 9/12 (75%) of total IBS patients, 5/6 (83.3%) of PI-IBS and 4/6 (66.6%) of idiopathic IBS patients. The total scores of the questionnaires showed a significant reduction from before FMT to 3 weeks after FMT as follows: IBS-SSS (326.6 ± 22.3 and 240.2 ± 33.6, respectively, *P* = 0.0009) and IBS-SQ (30.8 ± 3.3 and 11.6 ± 2.1, respectively, *P* < 0.0001). The scores of the different domains of IBS-SQ are shown in **Table 1**, which show a significant improvement in all of its domains except for anorexia.

**Table 1:** The scores of IBS-symptom questionnaire (IBS-SQ) domains in patients with irritable bowel syndrome.

Questionnaire	Before FMT	3 weeks after FMT	P-value
**Nausea**	3.7 ± 0.9	1.4 ± 0.6	**0.0025**
**Bloating**	7.9 ± 0.5	3.4 ± 0.8	**<0.0001**
**Abdominal pain**	6.3 ± 0.9	3.3 ± 0.8	**0.0012**
**Constipation**	4.3 ± 1.1	1.5 ± 0.7	**0.046**
**Diarrhea**	6.4 ± 0.9	1.2 ± 0.4	**<0.0001**
**Anorexia/loss of appetite**	2.3 ± 0.7	0.9 ± 0.4	0.087

Data are presented as the mean ± SEM. Comparison: Paired t test. FMT, fecal microbiota transplantation.

Significant differences are seen in the different domains (except for anorexia/loss of appetite) when comparing the symptoms of the patients before to after FMT. The bolded numbers are significant values <0.05.

### Gastroscopy and Immunohistochemistry

The duodenum of the patients was normal both endoscopically and microscopically. Msi-1 immunoreactivity was observed in both the cytoplasm and nucleus, and immunoreactive cells were found in the crypts of the duodenum. NEUROG3 immunoreactivity was found exclusively in the nuclei of cells that were observed in both the crypts and along side the villi. The immunoreactive enteroendocrine cells; CgA, secretin, CCK, GIP, somatostatin, and serotonin, were localized mostly in the crypts.

### Computerized Image Analysis

**Figure 1:**
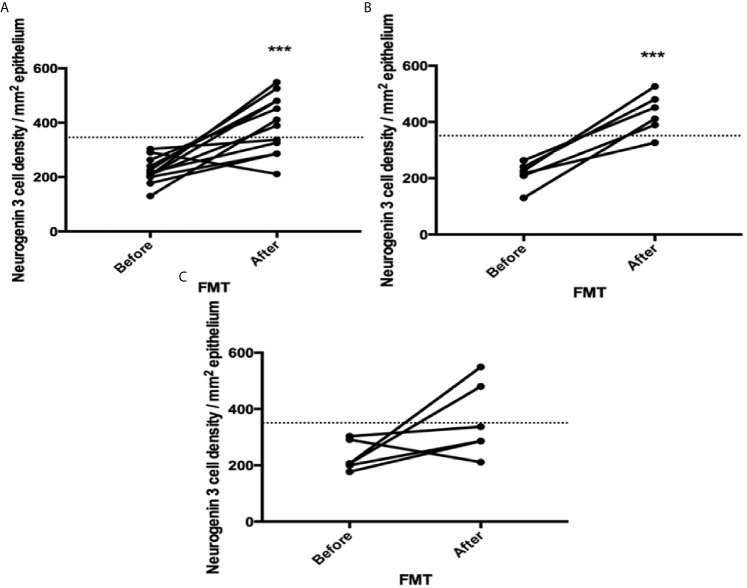
Neurogenin 3 cell densities before and after fecal microbiota transplantation (FMT) in **(A)** total, **(B)** post-infectious and **(C)** idiopathic irritable bowel syndrome patients. The dotted line represents the mean cell densities for the healthy control group. *** indicates *P* < 0.001.

**Figure 2:**
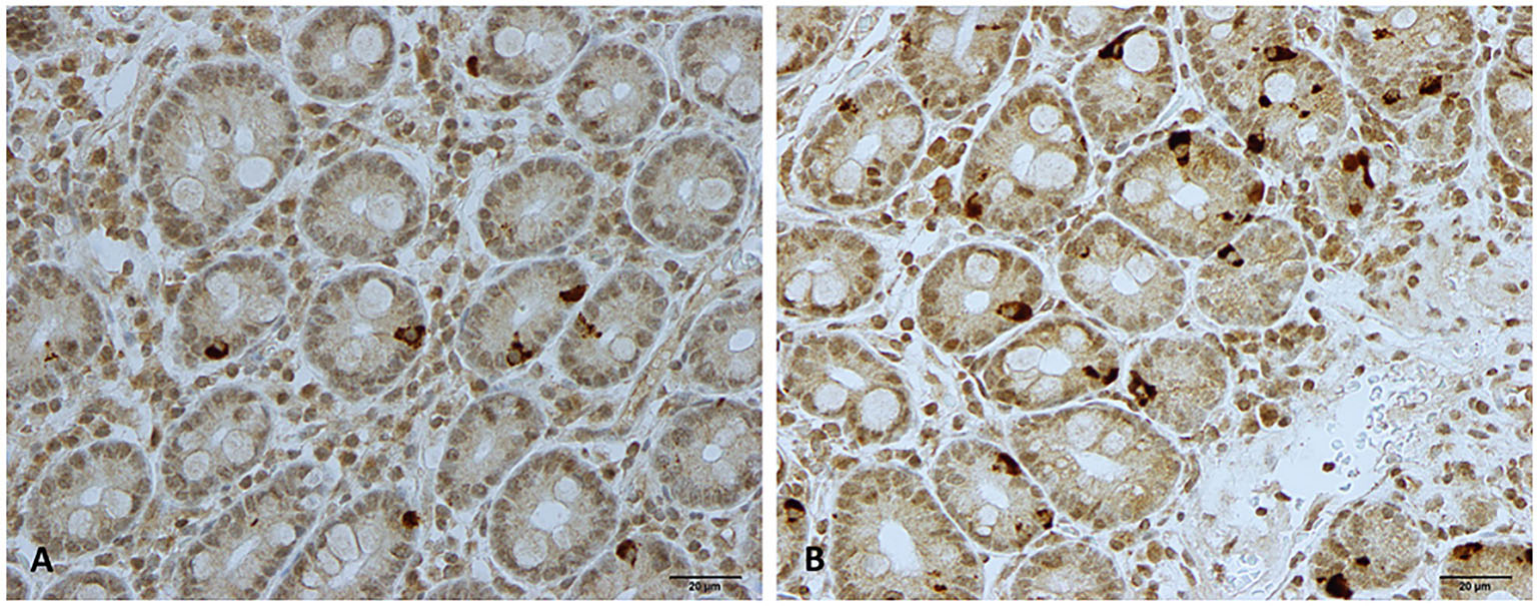
Neurogenin 3 immunoreactive cells in patients with irritable bowel syndrome **(A)** before and **(B)** after fecal microbiota transplantation.

**Figure 3:**
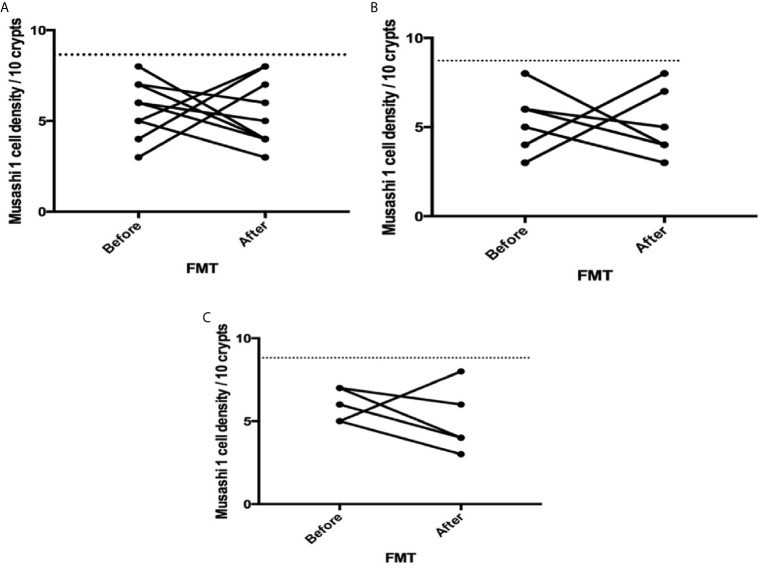
Musashi 1 cell densities before and after fecal microbiota transplantation (FMT) in **(A)** total, **(B)** post-infectious and **(C)** idiopathic irritable bowel syndrome patients. The dotted line represents the mean cell densities for the healthy control group.

**Figure 4:**
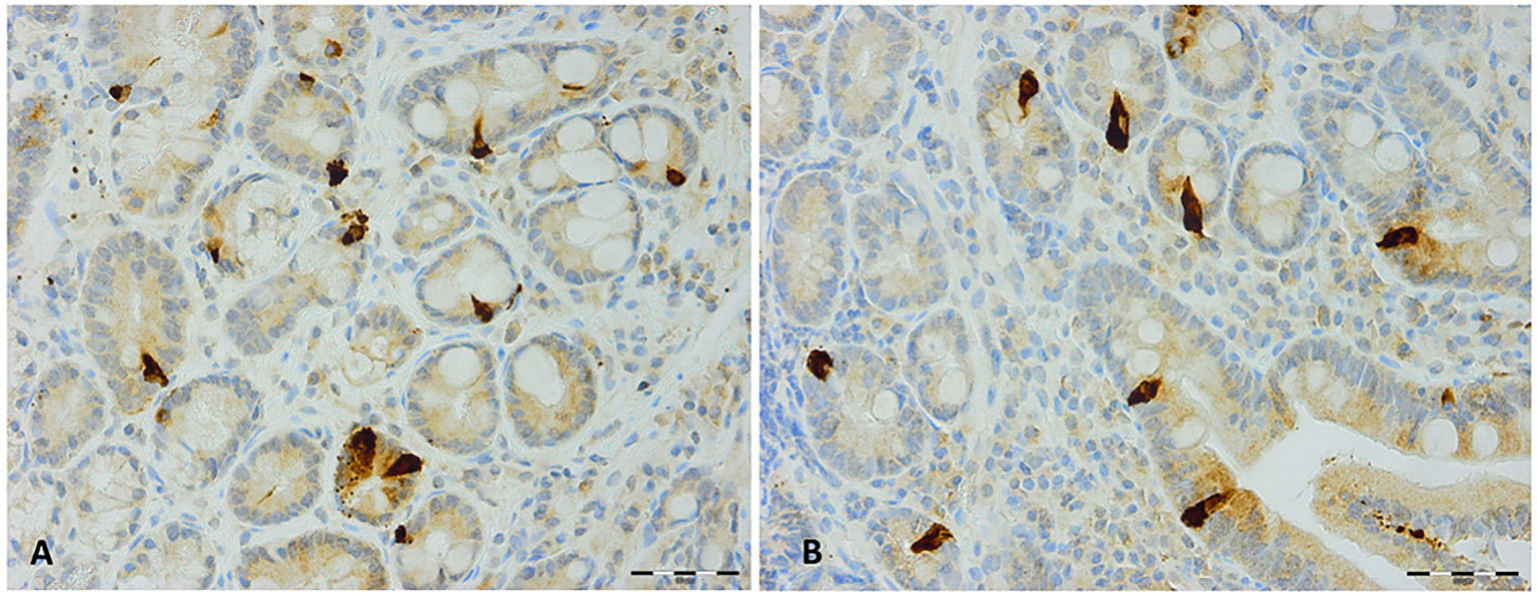
Secretin immunoreactive cells in patients with irritable bowel syndrome **(A)** before and **(B)** after fecal microbiota transplantation.

The densities of the stem cells and the enteroendocrine cells in the duodenum in controls and IBS patients are shown in **Tables 2** and **3**, respectively, before and after receiving FMT. The densities of the immunoreactive cells before and after FMT are shown in **Figures 1** and **2** for NEUROG3 and **Figure 3** for Msi-1. The cell densities of NEUROG3 were significantly lower (*P* = 0.04) for the total group of IBS patients before FMT than controls, but no significant difference was shown after FMT (*P* = 0.47). The cell densities of Msi-1 for the total group of IBS patients before and after FMT were significantly lower than the controls (*P* = 0.046 and 0.004, respectively). No significant changes were shown in the NEUROG3 and Msi-1 cells densities for both subgroups before and after FMT compared to controls (except for Msi-1 cell densities in idiopathic IBS after FMT, *P* = 0.02). When we compared cell densities for the patients before to after FMT, significant changes were seen in the cell densities of NEUROG3 densities for the total group of IBS and PI-IBS patients (*P* = 0.0006 and 0.007, respectively) but not for idiopathic IBS subgroup. No changes were seen in the Msi-1 cell densities when comparing IBS patients before and after FMT in all groups, **Table 2**. Several enteroendocrine cells showed significant differences in their cells densities compared to controls except for secretin (**Figure 4**) and GIP. The densities of the enteroendocrine cells increased in PI-IBS and decreased in the idiopathic IBS subgroup following FMT compared to before FMT. The directions of these changes were either towards or away from the values measured for controls, **Table 3**.

**Table 2:** Densities of stem cells in the duodenum of total IBS group, PI-IBS, and idiopathic IBS patients before and after receiving FMT.

Marker	Enteroendocrine cell densities (cells/mm^2^)							*^a^P*-value	*^b^P*-value	*^c^P*-value	*^d^P*-value	*^e^P*-value	*^f^P*-value	*^g^P*-value	*^h^P*-value	*^i^P*-value
	Control, *n* = 12	Total IBS,Before, *n* = 12	Total IBS,After, *n* = 12	PI-IBS, before, *n* = 6	PI-IBS, after, *n* = 6	Idiopathic IBS, before, *n* = 6	Idiopathic IBS, after, *n* = 6									
**Neurogenin 3**	340 ± 40	222.3 ± 13.8	394.3 ± 30.7	214.2 ± 18.5	430.5 ± 28.9	230.5 ± 21.5	358.2 ± 52.9	**0.004**	0.47	0.1	0.2	0.1	0.99	**0.0006**	**0.0007**	0.1
**Musashi 1**	8.75 ± 0.95	5.7 ± 0.4	5 ± 0.5	5.3 ± 0.7	5.2 ± 0.8	6 ± 0.4	4.8 ± 0.7	**0.046**	**0.004**	0.07	0.05	0.2	**0.02**	0.42	0.9	0.2

Data are presented as the mean ± SEM. Comparison: Kruskal–Wallis with Dunn’s post test, ^a^controls vs. total IBS patients before FMT, ^b^controls vs. total IBS patients after FMT, ^c^controls vs. PI IBS patients before FMT, ^d^controls vs. PI IBS patients after FMT, ^e^controls vs. idiopathic IBS patients before FMT, ^f^controls vs. idiopathic IBS patients after FMT; and Paired t test, ^g^total IBS patients before vs. after FMT, ^h^PI-IBS patients before vs. after FMT, ^i^Idiopathic IBS patients before vs. after FMT. FMT, fecal microbiota transplantation; IBS, irritable bowel syndrome. PI, post-infectious. The bolded numbers are significant values <0.05.

**Table 3:** Densities of enteroendocrine cells in the duodenum of controls, total IBS group, PI-IBS, and idiopathic IBS patients before and after receiving FMT.

Hormone	Enteroendocrine cell densities (cells/mm^2^)							*P^a^*-value	*P^b^*-value	*P^c^*-value	*P^d^*-value	*P^e^*-value	*P^f^*-value	*P^g^*-value	*P^h^*-value	*P^i^*-value
	Control, *n* = 12	Total IBS,Before, *n* = 12	Total IBS,After, *n* = 12	PI-IBS, before, *n* = 6	PI-IBS, after, *n* = 6	Idiopathic IBS, before, *n* = 6	Idiopathic IBS, after, *n* = 6									
**Chromogranin A**	228 ± 33.6	370.3 ± 21	269.8 ± 22	340.8 ± 34	422.7 ± 31	399.8 ± 20.9	316.8 ± 10.2	**0.0099**	**0.0133**	0.2	**0.006**	**0.005**	0.4	0.98	**0.0006**	**0.0065**
**Secretin**	74 ± 3.8	83.8 ± 4.9	86.7 ± 5.9	80.5 ± 8.8	89.7 ± 10.7	87.2 ± 4.8	83.7 ± 5.9	0.32	0.21	0.99	0.5	0.1	0.3	0.5	0.099	0.6
**Cholecystokinin**	80 ± 4.7	122.8 ± 6.7	110.7 ± 8.1	113 ± 10.4	126.5 ± 0.5	132.5 ± 7.2	94.8 ± 9	**0.0007**	**0.023**	0.055	**0.005**	**0.0008**	0.5	0.2	0.052	**0.0006**
**Gastric inhibitory peptide**	53.3 ± 5.7	65.1 ± 3.8	70.3 ± 6.2	60 ± 3 ± 3.7	84 ± 7.1	69.8 ± 6.3	57.2 ± 7	0.33	0.19	0.96	**0.01**	0.2	0.99	0.5	**0.014**	0.2
**Serotonin**	76.5 ± 13.6	135.1 ± 14.7	142 ± 12.8	100.5 ± 7.1	160.7 ± 16.6	169.7 ± 20.6	123.3 ± 17.3	**0.04**	**0.0088**	0.7	**0.005**	**0.01**	0.2	0.7	**0.012**	**0.034**
**Somatostatin**	42.3 ± 5.6	58.6 ± 4	66.2 ± 6.3	52.3 ± 2.7	78.5 ± 8.1	64.8 ± 7	53.8 ± 6.8	**0.033**	**0.012**	0.3	**0.006**	**0.03**	0.3	0.3	**0.011**	**0.017**

Data are presented as the mean ± SEM. Comparison: Kruskal–Wallis with Dunn’s post test, ^a^controls vs. total IBS patients before FMT, ^b^controls vs. total IBS patients after FMT, ^c^controls vs. PI IBS patients before FMT, ^d^controls vs. PI IBS patients after FMT, ^e^controls vs. idiopathic IBS patients before FMT, ^f^controls vs. idiopathic IBS patients after FMT; and Paired t test, ^g^total IBS patients before vs. after FMT, ^h^PI-IBS patients before vs. after FMT, ^I^Idiopathic IBS patients before vs. after FMT. FMT, fecal microbiota transplantation; IBS, irritable bowel syndrome; PI, post-infectious. The bolded numbers are significant values <0.05.

### Gut Microbiota

The analysis of the gut microbiota for the total group of IBS patients has been previously described (Mazzawi et al., 2018). In the current study, patients with PI-IBS before FMT had a significant increase in the bacterial signals for *Streptococcus* compared to donors at the beginning of the study and significantly reduced bacterial signals for *Megasphaera/Dialister, Actinobacteria*, and *Bifidobacterium* compared to donors at the beginning of the study, **Table 4**. Patients with idiopathic IBS before FMT had a significant decrease in the bacterial signals for *Firmicutes/Tenericutes/Bacteroidetes*, *Megasphaera/Dialister, Actinobacteria, Bifidobacterium*, and *Actinomycetales* compared to donors at the beginning of the study, **Table 4**.

**Table 4:** Fecal bacterial strains for donors at the beginning of the study and post-infectious IBS and idiopathic IBS patients before and after FMT.

Bacteria strain	Donors	Patients			*P*-values*	*P*-values**
			Before FMT	Week 3 after FMT		
**Post-infectious IBS**						
*Streptococcus*	47 (41, 59)	88 (59, 114)		79 (44, 210)	**0.038**	0.26
*Megasphaera/Dialister*	162 (58, 286)	19 (3, 57)		10 (5, 143)	**0.038**	0.11
*Actinobacteria*	160 (99, 321)	61 (21, 73)		219 (107, 435)	**0.0095**	0.9
*Bifidobacterium*	160 (96, 370)	62 (19, 86)		212 (92, 447)	**0.019**	0.9
**Idiopathic IBS**						
*Firmicutes/Tenericutes/Bacteroidetes*	260 (103, 475)	51 (24, 134)		**1**33 (14, 279)	**0.048**	0.18
*Megasphaera/Dialister*	4 (3.8, 247)	3.3 (3, 3.6)		3.1 (2.7, 98)	**0.048**	0.37
*Actinobacteria*	296 (251, 499)	89 (36, 115)		89 (44, 268)	**0.0025**	**0.02**
*Bifidobacterium*	358 (256, 645)	70 (38, 110)		100 (45, 282)	**0.0025**	**0.02**
*Actinomycetales*	15 (11, 18)	8.4 (8.1, 13)		13 (10,24)	**0.03**	0.95

Data are presented as the median (first, third quartiles). Comparison: Mann-Whitney U test. * donors compared to patients before FMT and ** donors compared to patients after FMT. FMT, fecal microbiota transplantation; IBS, irritable bowel syndrome.

The bolded numbers are significant values <0.05.

### Correlations

The correlation coefficient (r) and value of significance (*P*) for NEUROG3 cells with secretin were r = **−**0.89, *P* = 0.033 after FMT in the PI-IBS subgroup, and for NEUROG3 cells with serotonin were r = 0.89, *P* = 0.03 before FMT, and also with CCK were r = 0.89, *P* = 0.03 after FMT in the idiopathic IBS subgroup.

Certain strains of the gut microbiota correlated with stem and enteroendocrine cells. Before FMT, *Parabacteriodes* correlated with NEUROG3 cells in the total group (r = 0.76, *P* = 0.037) and with GIP cells in the PI-IBS group (r = 0.89, *P* = 0.03). In the PI-IBS subgroup, inverse correlations were found between *Streptococcus sanguinis and thermophiles* and the following: CgA (r = −0.94, *P* = 0.017), CCK (r = −0.89, *P* = 0.03), and serotonin (r = −0.89, *P* = 0.03) cells and between *Actinobacteria* and CgA (r = −0.89, *P* = 0.03), CCK (r = −0.94, *P* = 0.017) and serotonin (r = −0.94, *P* = 0.02) cells. After FMT, a correlation was found between NEUROG3 cells and *Actinobacteria* (r = 0.79, *P* = 0.025) also *Bifidobacterium* (r = 0.79, *P* = 0.025) in the total group of IBS patients and between GIP cells and *Actinobacteria* (r = −1, *P* = 0.017) also *Bifidobacterium* (r = −1, *P* = 0.017) in the idiopathic-IBS subgroup. In addition, correlations were found between *Lactobacillus* and NEUROG3 cells (r = 0.75, *P* = 0.038) in the total group of IBS patients and also somatostatin cells (r = 1, *P* = 0.017) in the idiopathic-IBS group. No correlations were found between Msi-1 and gut microbiota before or after FMT in any group of IBS patients.

The correlations between IBS symptoms using IBS-SQ questionnaire and the different enteroendocine cells were as follows: before FMT, pain correlated with the cell densities of each of CgA (r = 0.87, *P* = 0.02), serotonin (r = 0.87, *P* = 0.02) and CCK (r = 0.92, *P* = 0.01) and anorexia correlated with GIP (r = 0.91, *P* = 0.01) in the PI-IBS subgroup. In addition, bloating correlated with CgA (r = 0.85, *P* = 0.03), anorexia correlated with each of secretin (r = −0.91, *P* = 0.01) and GIP (r = −0.95, *P*=0.003), and nausea correlated with each of CCK (r= –0.89, *P*=0.01) and GIP (r= –0.95, *P*=0.003) in the idiopathic IBS subgroup. After FMT, diarrhea correlated with secretin (r = 0.83, *P* = 0.04) in PI-IBS and nausea with CCK cell density (r = −0.89, *P* = 0.02) in the idiopathic IBS subgroup.

### Adverse Events

No adverse events were reported during or following FMT.

## Discussion

In this study, the changes in IBS symptoms are associated with changes in the densities of the enteroendocrine cells and their progenitors NEUROG3 in the duodenum and in the gut microbiota of these patients 3 weeks after receiving FMT.

As previously reported (Mazzawi et al., 2018), approximately 75% of the total IBS patients showed improvement in their IBS symptoms following FMT. The densities of stem cells and enteroendocrine cells in the duodenum are abnormal in IBS patients before FMT, which is consistent with previous publications (El-Salhy et al., 2010; El-Salhy et al., 2014c; El-Salhy et al., 2015). Significant changes were mainly seen in the densities of NEUROG3 but not Msi-1 cells and all of the enteroendocrine (except for secretin) cells in IBS patients 3 weeks following FMT compared to before FMT. This indicates that the changes in the enteroendocrine cell densities are caused by a change in the differentiation of stem cells into enteroendocrine cells (as presented by NEUROG3). The observation that gut microbiota influence the differentiation of stem cells into enteroendocrine cells has been previously reported in the jejunum and colon of mice (Cani et al., 2007; Everard et al., 2011; Wichmann et al., 2013; Cani and Knauf, 2016). However, the exact mechanism explaining how the gut microbiota shape the fate of stem cells into enteroendocrine cells remains unknown.

A previous study showed that the CgA cell density, a common marker for endocrine cells (Deftos, 1991; Taupenot et al., 2003), correlated with NEUROG3 cell density in the small intestine of IBS patients (El-Salhy and Gilja, 2017). The correlations between the stem cells and enteroendocrine cells may be explained by that all of the enteroendocrine cells types arise from endocrine progenitors expressing NEUROG3 then the opposing activities of Pax4- and Arx-homeodomain transcription factors (Beucher et al., 2012). The inactivation of Pax4 leads to the up-regulation of Arx and the differentiation of these progenitors into different types of enteroendocrine cells and *vice versa*. Serotonin-producing enterochromaffin cells can differentiate in a NEUROG3 dependent and independent pathway. Similar to serotonin, somatostatin cells are generated from progenitors expressing NEUROG3 then Pax4. GIP-, secretin-, and CCK-cells arise from endocrine progenitors expressing NEUROG3 then Pax4 and Arx. The inactivation of Arx can cause these progenitors to be reallocated into the expression of somatostatin cells while GIP-, secretin-, and CCK-cells’ expression becomes impaired. However, an inactivation of Pax4 leads to an up-regulation of Arx; thus the differentiation of these progenitors into L cells (producing glucagon-like peptide-1 and peptide YY), while the differentiation of somatostatin-, serotonin-, and GIP-cells becomes impaired (Beucher et al., 2012). Therefore, the positive correlations between NEUROG3 cell densities and the different enteroendocrine cells along with the negative correlation between NEUROG3 cell density with secretin cell density, before and after FMT in the different IBS subgroups, may be attributed to the progenitor differentiation to other enteroendocrine cell types as previously explained. The densities of the enteroendocrine cells in the duodenum have changed 3 weeks following the FMT procedure. On one hand, the changes in the idiopathic-IBS patients are towards the healthy controls, which is similar to a previous study by our group using diet in managing IBS symptoms (Mazzawi and El-Salhy, 2017a; Mazzawi and El-Salhy, 2017b). On the other hand, the cell densities in PI-IBS patients increase away from the values measured for healthy controls, which may be due to the infectious origin of IBS in these patients (Dizdar et al., 2010).

The alterations in the strain signals of the gut microbiota have become normal after 3 weeks following FMT except for *Actinobacteria* and *Bifidobacterium*, which showed some changes towards normal but remained significantly different in the idiopathic IBS subgroup compared to the donors. The bacteria that correlated with the changes in the differentiation of stem cells into enteroendocrine cells as detected by NEUROG3 namely, *Parabacteroides*, *Actinobacteria, Bifidobacterium*, and *Lactobacillus*, also correlated with different enteroendocrine cells before and after FMT in the different groups. The changes in the bacterial strain signals occurred parallel to the changes in the differentiation progeny of stem cells and enteroendocrine cells densities, which indicated that gut microbiota might influence other enteroendocrine cell types than the previously reported enterochromaffin and L cells (Everard et al., 2011). This may also have shed some light on the mechanisms lying behind FMT and the positive effects of using these bacterial strains in the management of IBS symptoms (Whorwell et al., 2006; Drouault-Holowacz et al., 2008; Bixquert Jimenez, 2009; Sisson et al., 2014). The gut microbiota interact with the enteroendocrine cells through their microbial metabolites, short-chain fatty acids (especially butyrates) (Nohr et al., 2013; Cani and Knauf, 2016). Searching for a mechanism of action, we reasoned that because the vast majority of the gut microbiota are located in the ileum and in the colon and due to the paracrine and endocrine functions of the enteroendocrine cells throughout the gastrointestinal tract, the beneficial effects of FMT on the gut microbiota and their products (short-chain fatty acids mainly butyrate) (Mazzawi et al., 2019) might be related but not exclusive to this area of the gastrointestinal tract.

Visceral hypersensitivity plays an important role in the pathogenesis of upper gastrointestinal symptoms especially nausea, abdominal bloating and pain (Salet et al., 1998). Correlations of pain with serotonin and CCK cells can be explained since serotonin and CCK are involved in sensory and motor responses to distension in the intestinal tract and so perception of pain in IBS (Varga et al., 2004; Wendelbo et al., 2014). Although negatively correlated in this study, CCK is a satiety hormone and is involved in anorexia and nausea (Chua and Keeling, 2006; El-Salhy et al., 2014). GIP correlates negatively with anorexia (Stock et al., 2005) and does not induce nausea (Mentis et al., 2011). Secretin contributes to the rapid gastric emptying and pancreatic bicarbonate and fluid secretions therefore diarrhea in IBS (El-Salhy et al., 2014).

Two recently published meta-analyses have shown that fresh or frozen donor stool delivered *via* colonoscopy or nasojejunal tube may be beneficial in IBS (Xu et al., 2019; Ianiro et al., 2019). A newly published randomized, double-blinded, placebo-controlled study using a large cohort of IBS patients show that transplanting frozen stool *via* a gastroscope is an effective treatment for IBS patients (El-Salhy et al., 2019). In the current study, we have chosen to use gastroscopy because it is an easy and fast procedure and because patients with IBS often have small intestinal bacterial overgrowth, which would have escaped the suspension, had we chosen to use colonoscopy for FMT. The amount of transplanted fecal material, 30 g, used in this study is consistent with the published recommendations (Aroniadis and Brandt, 2013; Cammarota et al., 2014) before the starting point of the study in 2015 and with the European consensus on FMT (Cammarota et al., 2017). According to the newly published study (El-Salhy et al., 2019), both 30 g and 60 g FMT have shown significant responses compared to placebo in IBS patients.

No adverse events were noted following FMT in the current study despite previous data of minor adverse events that resolved spontaneously (El-Salhy and Mazzawi, 2018). We have previously published about the daily changes that occur to these symptoms during the first 3 days and 7 days following FMT, which showed a dramatic improvement in IBS symptoms shortly after FMT (Mazzawi et al., 2018). One can hypothetically assume that due to the severity of IBS symptoms before FMT, either that the patients did not relate to any worsening of their originally experienced bothersome symptoms or that they actually have benefited from the FMT with rapid improvement of the aforementioned symptoms.

The strength of the current study is the usage of validated methods to study the changes in the gut microbiota and immunoreactive cells’ densities and validated questionnaires to assess the changes in IBS symptoms. However, there are several limitations, such as the small number of the participants and the lack of a placebo-control group from which tissue biopsies could have been taken for the study of the stem and enteroendocrine cells. In spite of the small sample size of the different IBS groups, we found significant differences in the immunoreactive cells’ densities before and after FMT. However, further studies with larger groups of participants are required to be able to draw firm conclusions regarding the effect of FMT on the stem and enteroendocrine cells’ densities.

## Conclusions

Fecal microbiota transplantation improves the symptoms after 3 weeks in of IBS patients, both PI and idiopathic IBS. This improvement is associated with a change in the enteroendocrine cell density and the gut microbiota. The changes in the enteroendocrine cell density do not appear to be caused by changes in the stem cells or their early progenitors but rather by changes in the differentiation progeny as detected by changes in neurogenin 3.

## Data Availability Statement

The raw data supporting the conclusions of this article will be made available by the authors, without undue reservation.

## Ethics Statement

The studies involving human participants were reviewed and approved by the Regional Committee for Medical and Health Research Ethics in Western Norway (reference no.: 2013/1497). The patients/participants provided their written informed consent to participate in this study.

## Author Contributions

TM: included the patients, performed gastroscopies and FMT, quantified the enteroendocrine cells, drafted the manuscript, analyzed data, and planned the study. TM is a postdoctoral fellow, Helse-Vest (project number: 912309) www.helse-vest.no. ME-S: bought the necessary antibodies and covered all the expenses related to immunohistochemistry, quantified the stem cells, commented on the manuscript, analyzed data, and planned the study. GL: included the patients, performed gastroscopies and FMT, commented on the manuscript, and planned the study. TH: included the patients, performed gastroscopies and FMT, commented on the manuscript, and planned the study. All authors contributed to the article and approved the submitted version.

## Funding

This research was supported by grants to TH from Helse-Vest (grant no. 911802) and a grant to ME-S from Helse-Vest (grant no. 911976) and Helse-Fonna (grant no. 40415).

## Conflict of Interest

The authors declare that the research was conducted in the absence of any commercial or financial relationships that could be construed as a potential conflict of interest.

## Abbreviations

CgA, Chromogranin A; CCK, Cholecystokinin; FMT, Fecal microbiota transplantation; FODMAP, fermentable oligosaccharides, disaccharides, monosaccharides, and polyols; GI, Gastrointestinal; GIP, Gastric inhibitory peptide; IBS, Irritable Bowel Syndrome; PI, post-infectious; IBS-SQ, Irritable Bowel Syndrome Symptom Questionnaire; IBS-SSS, Irritable Bowel Syndrome Symptom Severity Scale; Msi-1, Musashi 1; NEUROG3, neurogenin 3; SEM, Standard error of mean.
